# Molecular Solar Thermal (MOST) Energy Storage—Definitions and Requirements Revisited

**DOI:** 10.1002/anie.202520673

**Published:** 2025-12-03

**Authors:** Dominic Schatz, Hermann A. Wegner

**Affiliations:** ^1^ Institute of Organic Chemistry Justus Liebig University Giessen Heinrich‐Buff‐Ring 17 35392 Giessen Germany; ^2^ Center for Materials Research (ZfM/LaMa) Justus Liebig University Giessen Heinrich‐Buff‐Ring 1 35392 Giessen Germany

**Keywords:** Molecular solar thermal energy storage, Mostophore, Photoswitches, Solar energy, Terminology

## Abstract

Molecular solar thermal storage (MOST) systems have gained increasing attention in the past years after an intense initial exploration phase around five decades ago. Over time multiple terms have been circulating in the literature, such as solar thermal fuels or solar thermal batteries. Besides clarifying the nomenclature, we introduce the term “mostophore” as a molecular entity able to harvest and store light energy in the form of heat. Furthermore, we revisit the requirements for practical systems and put the concept in a historic perspective with a future outlook.

## Graphical abstract

Molecular solar thermal storage (MOST) systems have recently regained great interest after a period of intense research around five decades ago. This perspective clarifies the nomenclature, and introduces the term “mostophore” for molecular entities that harvest and store light energy as heat. It revisits practical system requirements and provides a historical perspective with future prospects.

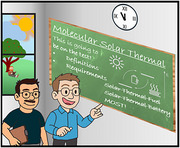

## 1 Harvesting Light—Storing Heat

The sun provides energy enough to support the worlds energy needs.^[^ ^1^ ^]^ However, the offer and demand does not always coincide in time and location. Fossil resources are still used to compensate times of large energy demand and low renewable energy production. These fuel reserves are limited and contribute to the man‐made climate change. This challenge is addressed with storage solutions that can be charged using a surplus of energy during high production/low demand periods and deliver it during low production/high demand periods. We as a society require mostly two energy types: electrical and thermal energy.^[^ ^2^ ^]^ Therefore, storing solar energy, directly in the energy form later needed, can minimize tedious and inefficient energy conversion. While electricity might be of “a higher quality” and more versatile form of energy, heat is the by far most needed.^[^ ^2^, ^3^ ^]^ The sun, as the most abundant renewable energy source, offers multiple ways to generate energy in a variety of forms and qualities. The most researched and established technology, solar cells, can generate useable electricity due to the photoelectric effect of semiconductors during irradiation. The thereby generated electricity needs to either be consumed straightway or stored externally in a battery. Producing and storing electrical energy during irradiation can also be condensed into a single component by utilizing photoelectrodes.^[^ ^4^ ^]^

**Figure 1:**
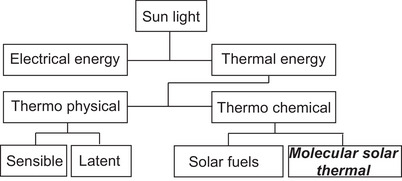
Solar energy can be stored as either electrical or thermal energy. Thermal energy storage methods include physical heat storage, such as sensible (insulation required) and latent heat storage (phase change, e.g., solid to liquid). Alternatively, thermochemical storage involves creating high‐energy compounds (solar fuels like hydrogen) for exothermic reactions or transforming molecules into metastable states in molecular solar thermal (MOST) energy storage.

For the direct harvesting of thermal energy of sunlight, a plethora of technologies is available (Figure 1).^[^ ^5^ ^]^ However, the direct saving of heat as sensible heat for later use requires insulation to impede dissipation of the energy.^[^ ^6^ ^]^ The most convenient liquid material for this application is water, which has a high specific heat capacity and low cost and toxicity. While this is a viable strategy for decentralized, daily storage, heat losses make this strategy unsuitable for seasonal applications. A similar strategy involves storing of solar heat as latent heat dictated by an isothermal phase transition during the energy uptake.^[^ ^7^, ^8^ ^]^ Alternatively, the energy of the sun can be converted into chemical energy. One option is the promotion of a chemical reaction to create a high energy compound, such as hydrogen or hydrocarbons, which can be converted in a separated process to heat and side products.^[^ ^9^, ^10^ ^]^ Here, the energy is stored in the enthalpically unfavorable material, which itself needs to be contained in a tank, until the energy is needed. A significant downfall of this technologies is that different compartments for the synthesis of the high energy compound, the storing of the compound, and also for the heat generation are necessary.

**Figure 2:**
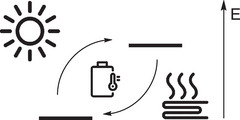
The MOST concept: The sun's energy is stored by converting a molecule from a ground state to a higher energy, meta‐stable state. Ideally, the molecule can stay indefinitely in this state before being converted back to the ground state by a trigger, while releasing the excess energy in the form of heat.

An alternative very attractive concept relies on the reversible conversion of a molecule to a metastable higher energy state upon irradiation (Figure 2).^[^ ^11^, ^12^ ^]^ With an external trigger the compound can be converted back to the ground state liberating the energy as heat. This technology excels as the molecules employed harvest and store the energy at the same time, allowing a closed system in simple and self‐contained set‐up.^[^ ^13^ ^]^

## 2 MOST—The Name Game

**Figure 3:**
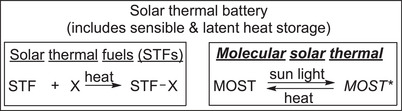
Differentiation of the terminology for heat storage: Solar thermal fuels (STFs) describe sun‐generated compounds, which can be converted from a stable/persistent entropically disfavored compound to heat upon reaction with an auxiliary. Molecular solar thermal (MOST) storage systems describe molecules, which can be reversible converted to a storage state and back while releasing heat. All technologies storing and releasing heat generated from the sun can be summarized as solar thermal batteries.

The first idea toward MOST has been already formulated more than a century ago by Weigert, who worked on the thermodynamic explanation of the anthracene dimerization.^[^ ^14^ ^]^ He introduced the concept of an “umkehrbaren photochemischen Reaktion” (german for: reversible photochemical reaction), in which a photochemically induced state relaxes in the dark back to the ground state.^[^ ^15^ ^]^ Since Weigert's report multiple names circulate for this technology, such as molecular solar thermal storage (MOST) systems, solar thermal fuels (STFs), or solar thermal batteries (STBs) (Figure 3). Although these terms are often used interchangeable to describe the storage of photon energy as chemical energy, we consider specific differences between these concepts and propose the following terminology: The thermal energy generation from solar irradiation can be distinguished based upon a photophysical or photochemical storage mechanism. The photophysical concepts explore storage materials that rely on a phase change, or store sensible heat during the energy uptake. Thermochemical storage materials can be divided into solar (thermal) fuels, or into MOST materials. Fuel, in the context of energy delivering, is defined by the Cambridge Dictionary as “a substance that is used to provide heat or power, usually by being burned.”^[^ ^16^ ^]^ The category of solar fuels therefore contains the compounds that are stable/persistent under ambient conditions but are consumed upon reaction with an auxiliary (e.g., oxygen) upon energy delivery. Possible technologies rely on an artificial photosynthesis by splitting water via photocatalysis or carbohydrates. We think that using this term for a chemical storage application is in agreement with the opinion stated by Aprahamian and Goldup regarding the usage of “fuel” in non‐equilibrium states.^[^ ^17^ ^]^ The term solar fuels differentiates them from molecular solar thermal storage material, which themself rely on the reversibility of a ground state and a higher energy isomer. These compounds can absorb light of a specific wavelength, perform a photoisomerization during which the absorbed energy, either in the form of bond energy (in case for pericyclic isomerizations) or in form of strain (in case of configurational isomerization). Upon reisomerization, the chemical energy is released as useable heat. The MOST concept therefore is based on the formation of metastable isomer, which fully regenerates to deliver the stored energy.

The last circulating term used to describe MOST compounds is solar thermal batteries. The term battery is unambiguously connected with electrochemical storage (definition Cambridge dictionary: “a device that produces electricity to provide power for electronic devices, cars, etc.”).^[^ ^18^ ^]^ We, therefore, suggest that the technologies of MOST materials and solar fuels should not be used synonymous with solar thermal battery. If the term battery is used nevertheless, we recommend to only using it to refer to complete devices, including all technologies storing heat generated from the sun, and not principles, compounds, or materials.

Since the introduction of MOST systems, a variety of photoswitchable molecules have been examined, studied, and optimized for energy storage purposes with each having its own advantages and disadvantages.^[^ ^11^ ^]^ Herein, we introduce the term “mostophore” similar to the term “chromophore” (ancient Greek: χρώμα = color + φορός = carrier). A mostophore is capable of being transformed by absorbing light into a metastable higher energy state. Upon an external stimulus, it can be converted back to the ground state. It represents the core structure with a specific functional unit, which can be altered by substituents, can be incorporated into larger ensembles or categorized according to its performance. We advocate that a distinct definition of such a subunit, which can strategically be added onto a molecular scaffold to open it up to solar thermal storage solutions, will facilitate navigation the vast amount of literature about chromophores, molecular machines, photoisomers, and solar fuels, by naming a motif directly after its intendent use as a storage solution.

“*A molecular entity, which is able to be converted from a low energy structure to a meta‐stable isomer upon irradiation, and can release the energy in the form of heat upon a trigger is called a* ***mostophore*** *(MOST = Molecular Solar Thermal).”*

**Figure 4:**
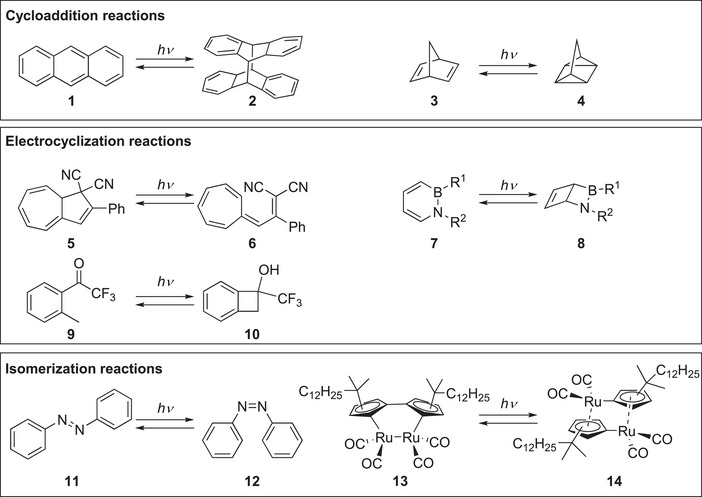
Classification of mostophores according to their reaction mechanism: The dimerization of anthracene **1** and the intramolecular reaction of norbornadiene **3** rely on cycloaddition reactions. Electrocyclization reactions are responsible for the MOST activity of dihydroazulene **5**, azaborinine **7,** and the ketone **9**. Azobenzenes **11** and the ruthenium complex **13** are based on isomerizations.

Transformations utilized in this context are pericyclic reactions (cycloaddition reactions, electrocyclizations) or isomerizations [interconversion of (*E*)↔(*Z*) or constitutional isomers], which are reversible (Figure 4). The initial example by Weigert, the dimerization of anthracene (**1**) is based on a [4 + 4] cycloaddition reaction. Han and coworkers recently presented an impressive example of self‐catalyzed heat release based on such as system.^[^ ^19^ ^]^ Also, the probably most advanced mostophore system, norbornadiene (**3**) – quadricyclane (**4**) is based on a [2 + 2] cycloaddition.^[^ ^20^ ^]^ Around 20 years ago, Brøndsted Nielsen and coworkers introduced the dihydroazulene **5** – vinylheptafulvene **6** system as a mostophore featuring a transparent storage state, which is based on an electrocyclization reaction.^[^ ^21^ ^]^ Very recent mostophore systems, such as the azaborinine **7** and its Dewar variant **8** by Bettinger and coworkers,^[^ ^22^ ^]^ as well as the cyclization of the quinodimethane derived from the ketone **9** by Wahl and coworkers utilize also an electrocyclization as key transformation.^[^ ^23^ ^]^ Furthermore, isomerization reactions, such as the (*E*)↔(*Z*) isomerization of azobenzenes **11**/**12** or the isomerization of the (fulvalene) diruthenium compound **13**/**14**, serve as basis for mostophore.^[^ ^24^ ^]^ A detailed discussion of the advantages and disadvantageous of these mostophores can be found in recent reviews.^[^ ^11^, ^25^, ^26^, ^27^, ^28^ ^]^

## 3 Performance Parameters of a Mostophore

**Figure 5:**
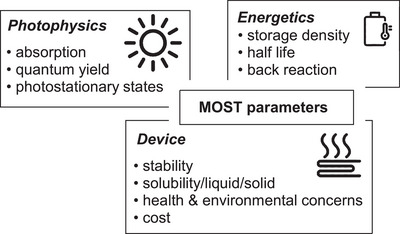
Performance parameters of a system can be divided in three categories: Photophysics, energetics, and devices.

For classification and comparison, a definition of important parameters for MOST storage would be highly desirable. Such MOST parameters can be divided into three categories (Figure 5):

- ***Photophysical properties***: Absorption (solar spectrum match, difference between the photoisomers) quantum yield of the isomerization, obtainable photostationary states, and a solar conversion efficiency^[^ ^29^ ^]^
- ***Energetical properties***: The activation barrier of the thermal back isomerization, energy storage capacity based on molecular weight, and energy difference between stable and metastable state, and possibilities to trigger the heat release
- ***Device properties***: Stability, solubility (in benign solvents) or viscosity, pumpability, controlled, non‐explosive energy release, health and environmental concerns, cost, and scale of production

What makes a “good” mostophore? In principle, any molecule, which can be transformed via irradiation from a lower energy form into a metastable higher energy form can be utilized for MOST application. Most properties of a specific mostophore are intertwined, though, depending on each other and challenging to optimize without negatively influencing other parameters. For example, the absorption maxima of mostophores are bathochromically shifted by the introduction of both an electron donor and acceptor substituent to establish a conjugated push‐pull system.^[^ ^30^ ^]^ However, the resulting compounds usually have very low thermal half‐lives.^[^ ^31^ ^]^ As another example, while the thermal half‐life of an azobenzene can be improved by stabilizing the (*Z*)‐AB isomer, this change will negatively influence the energy density given by the (*E*)‐(*Z*) energy difference.^[^ ^32^ ^]^ Depending on the envisioned application, the focus on the most important parameter can shift. A solid photoswitch, for example, can be of interest as a MOST material for surface materials,^[^ ^33^, ^34^ ^]^ while a low viscosity switch can be applied for large‐scale pumpable storage.^[^ ^35^ ^]^ If a solvent is required, water represents a sustainable option.^[^ ^36^, ^37^ ^]^ However, its high heat capacity can complicate the heat release. The required half‐lives can vary from seasonal to daily storage applications. Some of these parameters though should be optimized regardless of the intended use case.

### 3.1 Energy Density

**Figure 6:**
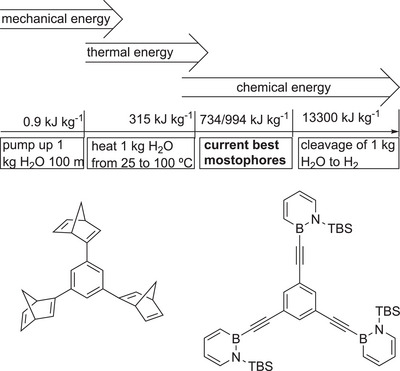
Energy density of current mostophores in view of different storage strategies, such as mechanical, thermal, and chemical energy. Although mostophores store thermal energy, it is based on bond breaking/forming processes characteristic for chemical storage. TBS = *tert*‐butyldimethylsilyl.

The energy density can be listed as one key parameter and should hold a direct comparison to other storage systems. Three different concepts for energy storage can be distinguished: Mechanical, thermal and chemical (Figure 6). Mechanical energy can be reflected by the energy needed to pump 1 kg of water to a height of 100 m: E = m * g * *h* = 981 *J* = 0,0003 kWh; thermal energy can be illustrated in the amount of energy needed to heat water from 25 °C to 100 °C: E = m * c_V_ * Δ*T* = 315 kJ = 0,087 kWh; chemical energy (energy in chemical bonds) is demonstrated in the cleavage of 1 kg of water to produce 111 g of H_2_: E = ΔG°_m_ = 13.300 kJ = 3,7 kWh. The examples show that between the different concepts a rough difference of two orders of magnitude in energy density exists, rendering chemical storage as the densest storage medium. The MOST concept can also be seen in this regard as the bonding situation is changed by irradiation.

In a seminal review by Yoshida an energy density of 100 kcal kg^−1^ has been stated as minimal energy density.^[^ ^38^ ^]^ An even lower value of 300 kJ kg^−1^ has been put forward by Bren and coworkers in 1991,^[^ ^39^ ^]^ frequently circulating in the MOST community. As an example, in the same year that Bren stated his energy requirement for a thermal storage device, Sony announced the first commercialized lithium‐ion battery.^[^ ^40^ ^]^ This novel electrochemical cell had an energy density of 80 Wh kg^−1^ or around 290 kJ kg^−1^. Obviously, the storage density of lithium‐ion batteries skyrocketed in the following years with the change of active electrode materials. It became apparent that parameters like charge/discharge performance and especially the volumetric energy density is more important for consumer products. While there are battery applications that claim to deliver up to 2.6 MJ kg^−1^ or 6.0 MJ L^−1^,^[^ ^41^ ^]^ the current Tesla batteries show an energy density of 241 Wh kg^−1^ corresponding to 868 kJ kg^−1^.^[^ ^42^ ^]^ Interestingly, for ion batteries the final device, containing redox active material, electrodes, electrolyte, membranes and hull were considered in the gravimetric density. In contrast, the energy density of MOST storages systems (molar or gravimetric) is usually referenced only to the active mostophore material without considering the necessary infrastructure, such as pumps, etc. or even the solvent.

Nevertheless, a comparison of MOST storage materials to a technology, that focuses on compact storage devices (e.g., handheld, or car batteries), might not be suitable. A more reasonable electrochemical storage system would be redox flow batteries (RFBs), as they show engineering similarities to MOST devices.^[^ ^43^ ^]^ First, the capacity and power are decoupled from another, as the electrode stack and the storage tank are separate compartments, similar to a MOST storage and a charging irradiation unit, and a discharging heat exchanger/catalyst unit. Second, the application for redox flow batteries is large scale, grid stabilizing (and maybe even decentralized) storage, which can also be envisioned for MOST storage solutions. However, RFBs still require a collector system to harvest the solar energy. For a vanadium based redox flow battery with 2.5 M redox active material, an energy density of 140 kJ L^−1^ or 220 kJ kg^−1^ (calculated for VSO_4_), can be calculated.^[^ ^44^ ^]^ These values are well within the reach of current discussed MOST materials.

A more ambitious thermal storage density is given by Prasher et al. where a gravimetric energy density of > 1 MJ kg^−1^ with a volumetric density of > 0.1 MJ L^−1^ is promoted to have an improvement from thermochemical (high gravimetric, low volumetric), and thermophysical (low gravimetric, high volumetric) storage solutions.^[^ ^13^ ^]^

The best MOST systems currently are very close to the value of 1 MJ kg^−1^, such as tris‐dihydoazaborinine reported by Bettinger.^[^ ^45^ ^]^ The combination of multiple mostophores in one molecules shows great promise.^[^ ^26^, ^46^ ^]^ For example, a trisnorbornadiene derivative shows a storage density of 0.734 MJ kg^−1^.^[^ ^47^, ^48^ ^]^ Interference in absorption, energy transfer between the mostophores and other interactions have to be considered in the design when combining multiple mostophores. In combination with sensitization system, which could be converted in sunlight with maximum energy storage efficiency of 5.8 % was presented. In 2011 Boulatov and coworkers calculated a maximum energy density for MOST systems of 1.7 MJ kg^−1^ for a mostophore with a molecular weight of 100 g mol^−1^.^[^ ^49^ ^]^

### 3.2 Light Energy Input

**Figure 7:**
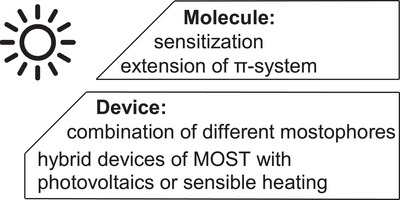
Strategies to improve the utilization of the sun's energy. On the molecular level sensitization is the preferred strategy. Alternatively, absorption of longer wavelengths can be achieved by extending the π‐system. Additionally, devices can be optimized by stacking multiple flow collectors or combination in hybrid devices with other solar harvesting technologies.

The ideal mostophore absorbs across the whole spectrum of the sun. In reality, this poses a challenge, as especially small ring systems, such as the naked NBD, only interacts with UV‐light.^[^ ^20^ ^]^ Early on, there have been strategies to tackle this challenge to also allow energy input from longer wavelengths (Figure 7). One option, the extension of the π‐system by attaching suitable substituents has been successfully demonstrated.^[^ ^50^ ^]^ However, this structural modifications usually go hand in hand with an increase in molecular weight, which equals to lower gravimetric energy density and reduced half‐life. Already in fundamental studies of Hammond and coworkers sensitization has been shown to be effective.^[^ ^51^ ^]^ Recently, Kerzig and coworkers presented a system with an overall energy efficiency of 5.8% using a NBD‐derivative in combination with a suitable sensitizer.^[^ ^47^ ^]^ Hence, sensitization represents the a versatile and efficient approach. It should be mentioned, though, that this approach can likely not be combined with the introduction of donor or acceptor substituents to the mostophores as a result of potential electron transfer side reactions. Besides the absorption, the excited state dynamics that compete with the desired photoreaction, such as emission, intersystem crossing (ISC), proton/electron transfer etc., have to be considered. These processes can drastically reduce the efficiency of a mostophore. Additionally, concentration dependence can be important in case of intermolecular MOST processes, e.g. for the anthracene mostophore.

There is also the possibility to improve this MOST‐parameters via device engineering to make better use of the sun's energy.^[^ ^52^ ^]^ For example, the stacking of flow systems with multiple mostophores with different absorption profiles allows to capture a broader range of the solar spectrum.^[^ ^53^ ^]^ An additional option is the combination with other sun energy harvesting technologies, such as water heating^[^ ^54^ ^]^ or photovoltaics.^[^ ^55^ ^]^

### 3.3 Heat Output

**Figure 8:**
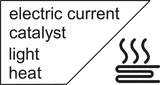
The heat release can be triggered by different stimuli, which are highly dependent on the intended application and the MOST systems.

Besides the efficient energy input, the release of the energy at a given time is also crucial (Figure 8). The simplest approach relies on a suitable half‐life to realize for example a day‐night cycle, where during the day the MOST system is charged, while it releases the energy overnight. However, much more versatile is the release on demand by an external trigger, which is particularly important for long‐term energy storage, such as a seasonal cycle with energy harvesting during summer and release during winter. As the half‐life is temperature dependent a sudden increase of temperature can stimulate the heat output. Han and coworkers recently reported a self‐catalyzed heat release system based on anthracene.^[^ ^19^ ^]^ This has the advantageous that a short laser pulse is enough to release the whole energy of the system. A step‐wise controlled release, however, is in this case not possible. Also, light can be used as stimuli to trigger the heat release.^[^ ^35^, ^56^ ^]^ While very well controllable, it uses the same input for control as for the energy input, making this approach less versatile.

The use of a catalyst has been shown basically for all mostophores to efficiently release the stored energy. This requires the contact of the mostophore with the catalyst for a short period of time, usually realized via flow set‐ups. Alternatively, the catalyst has been incorporated in magnetic nanoparticle which can be easily removed from the system.^[^ ^57^ ^]^ One of the most elegant solutions is the use of an electric current.^[^ ^58^ ^]^ This can be easily controlled.^[^ ^59^ ^]^ For the azobenzene mostophore, it has been shown that the heat release can be catalyzed by electrons (and holes), which again poses challenges in the controllability.^[^ ^60^ ^]^ As can be seen, the heat release very much depends on the intended application of the MOST system as well as on the mostophore utilized. Hence, this aspect also has to be optimized in close monitoring with the other parameters.

Obviously, the other parameters, such as efficiency of the photoisomerization, which correlates with, e.g., the quantum yield and obtainable photostationary state or stability are crucial for pushing the MOST technology toward real world applications.

In the end, it is the economic factor, which determines the breakthrough. Although the MOST technology development is still in an early‐stage comparison of this aspect will be done to the already mentioned redox flow batteries: Ferret et al. stated in their review about the perspective of redox flow batteries, a targeted cyclic stability of 10 000 cycles, and an economic limit of 0.05 € kW‐1 h‐1 cycle‐1 for a sustainable energy storage solution.^[^ ^44^ ^]^

## 4 Past, Now, and Future of MOST

**Figure 9:**
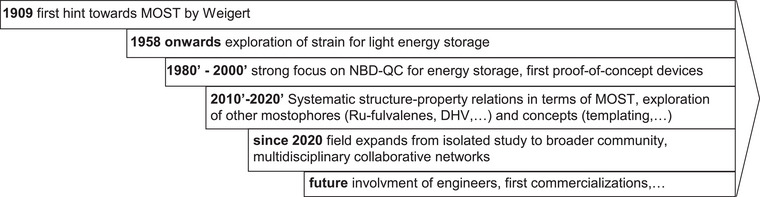
Development stages of the MOST field since its initial proposal by Weigert in 1909 from isolated studies to a vibrant research community.

Although Weigert hinted towards light energy storage in molecules already in 1909, the MOST field has been dormant for decades (Figure 9). About five decades later Splitter and Calvin suggested small ring systems with their inherent strain for the same purpose.^[^ ^61^ ^]^ At that time also the NBD‐QC was popularized as mostophore.^[^ ^62^ ^]^ In 1980, Yoshida and coworkers took the MOST idea from a conceptual proof‐of‐concept studies to reality by demonstrating the first MOST device based on the NBD‐QC mostophore.^[^ ^63^ ^]^ He suggested the term “molecular energy storage system for solar energy” for the concept. Despite the success, research activity in MOST declined in the following decade. Only around the 2010′ research interest sparked again, fueled by a comprehensive review by Boulatov.^[^ ^49^ ^]^ Since then, the other mostophores were introduced, as well as general concepts to increase MOST performance, e.g., via templating.^[^ ^64^ ^]^ Also, systematic optimization of the properties of mostophores has been pursued. In the last decade, the MOST concept has gained more and more popularity also across disciplines such as organic chemistry, spectroscopy, photochemistry, and theoretical chemistry. New, highly potent mostophores have been presented, such as the azaborinines^[^ ^22^ ^]^ or trifluoromethylarylketones.^[^ ^23^ ^]^ These efforts are also reflected in larger research consortia, such as the MOST project within the Horizon 2020 funding scheme^[^ ^65^ ^]^ or the FORMOST DFG Research Group on “Molecular Solar Energy Management – Chemistry of MOST Systems.”^[^ ^66^ ^]^ Besides the progress made in the area, the first commercial MOST device is still waiting to be launched. Therefore, the involvement of engineers is proposed as the next level to push the MOST from the research labs to the day‐to‐day life.

## 5 Conclusion

The MOST concept features unique advantages to all other energy storage solutions:

- It is a closed system: One molecule acts as energy harvesting and storage entity. This is in contrast to photovoltaics. Here, the solar panels produce the energy, and the storage, however, has to be done in battery. In solar fuels, a chemical compound is produced with sunlight. However, upon energy release the compound is transformed to a very different composition. Therefore, also mobile applications can be envisioned.
- Storage times can be adjusted to the need without the necessity of insulation; the current state of the art heat storage of the sun's energy utilizes the heating of water. While this system excels with its price and simplicity, insulation is needed determining the storage duration. The maximal storage density is defined by the heat capacity.

After a high time of MOST research in the 1960′–1990′ the field was reinvented with the increasing heat in the debate about shortage of fossil fuels and climate change. Especially in the past decade, very promising contributions in molecular design and overall strategy have been presented. However, a suitable mostophore is only one requirement for a successful application. Now, device design and operational readiness have to be ensured. While other reviews highlight design strategies and performance of mostophores,^[^ ^11^, ^12^, ^25^, ^27^, ^28^, ^52^, ^67^, ^68^, ^69^ ^]^ this perspective aimed to clarify terms and background to further unify research efforts and spark interest in the field by other researchers across disciplines.

## Conflict of Interests

The authors declare no conflict of interest.
